# Early repolarization pattern with oral liquid nicotine

**DOI:** 10.1186/s12872-025-05170-0

**Published:** 2025-09-08

**Authors:** Julian Wolfes, Lea Reitnauer, Lars Eckardt

**Affiliations:** 1 Department of Cardiology II (Electrophysiology), University Hospital Münster, Albert-Schweitzer-Campus 1, Münster, 48149 Germany; 2 Department of Medicine A, Hematology, Oncology and Pneumology, University Hospital Münster, Albert-Schweitzer-Campus 1, Münster, 48149 Germany

## Abstract

While most sudden cardiac deaths are due to structural heart disease or cardiac ischemia, intoxications are rather rare and often unrecognized. Here we present a case of a 35-year-old patient who trickled cumulative 60 mg of the pure nicotine liquid. This led to cardiac arrest and ventricular fibrillation. After defibrillation the ECG showed pronounced early repolarization pattern with an AV block I°.

## Background

While most sudden cardiac deaths are due to structural heart disease or cardiac ischemia, intoxications are rather rare and often unrecognized. Against this background long-term side effects of smoking are well known, while acute toxic effects are not as well characterized.

### Case presentation

We report a case of oral nicotine intoxication with subsequent resuscitation. We saw a 35-year-old patient with an unremarkable own and family history. After 4 pack years of smoking he had ceased smoking in spring 2023 and thereafter occasionally consumed e-cigarettes. On New Year´s Eve 2023 he attended a party with his wife. To relieve nicotine withdrawal symptoms, he trickled cumulative 60 mg of the pure nicotine liquid (20 mg nicotine/ml) in his mouth mucosa in the early morning of January 1st. 30 min later the patient suddenly complained of palpitations, became unconscious, and required resuscitation. Following bystander chest compressions the first emergency ECG showed ventricular fibrillation, which was successfully defibrillated. The patient was intubated and brought to our Cardiac Arrest Center.

**Fig. 1:**
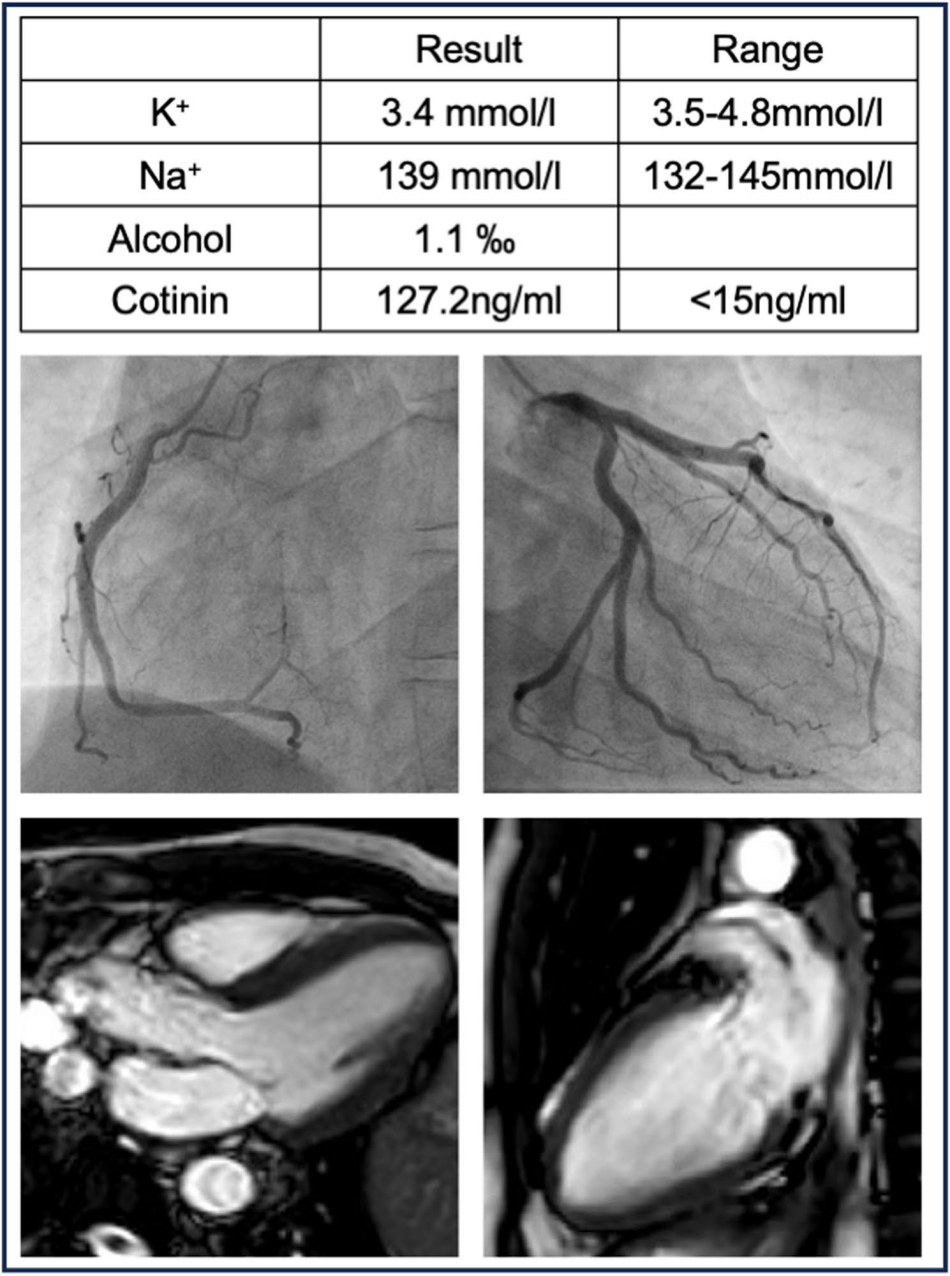
Patient´s laboratory findings (upper row), coronary angiography (middle) and cardiac MRI (bottom)

On admission, the patient’s first 12-lead ECG showed an early repolarization pattern with an AV block I° (PR interval of 240ms). First-degree AV block regressed rapidly, while the early repolarization pattern increased within 24 h before gradually regressing as shown in (Fig. 1)without disappearing completely. A coronary angiography excluded coronary heart disease. The patient was extubated without neurological impairment, cardiac MRI, thoracic and cranial CT were performed, which also showed no significant findings.

**Fig. 2:**
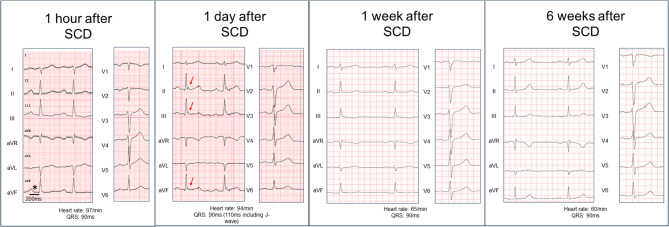
Changes in the 12-lead-ECG following nicotine intoxication and sudden cardiac death (SCD). Asteriks indicate prolonged PR interval. Red arrows indicate prominent early repolarization pattern. Paper speed: 50mm/s, voltage: 10mm/mV

Apart from a blood alcohol level of 1.1‰, the usual laboratory values were within the normal range. The level of the nicotine metabolite cotinine was 127.2 ng/ml (< 15ng/ml) in the gas chromatography-mass spectrometry, significantly above the usual limit. The remaining toxicological screening was negative (Fig. 2). In the course of the inpatient stay and under nicotine abstinence, the ECG changes regressed. Similarly, no arrhythmias were documented. Despite the nicotine-induced early repolarization syndrome with a most likely drug-induced resuscitation the patient and his wife decided on prophylactic therapy with a subcutaneous ICD. The patient was discharged after 8 days of inpatient monitoring. There were no new arrhythmias under nicotine-free conditions during follow up.

## Discussion

While the long-term effects of cigarette and e-cigarette use on the cardiovascular system are well known, the short-term effects of nicotine and its metabolites are much less studied and understood. Intoxication with nicotine can lead to ganglionic blockade with hypotension, bradycardia, and unconsciousness [1]. Although nicotine is one of the most potent poisons of all and its intoxication can be fatal via respiratory failure or circulatory collapse [2], lethal intoxications are rare [3]. The lethal dose of nicotine is reported to be 0.5-1.0 mg/kg [3], so in our patient with a body weight of 72 kg a potentially lethal dose was ingested.

The electrophysiological mechanisms by which nicotine can cause ventricular fibrillation are insufficiently studied. Interestingly, changes in AV time and early repolarization syndrome were also seen in the ECGs of mice exposed to e-cigarette smoke by Carll et al. [4], where the main mechanisms are probably based on interactions with the transmisson in the autonomic nervous system [5]. The oral mucosa intake most likely resulted in a sudden increase of serum nicotine with subsequent ventricular fibrillation in an otherwise healthy young man with a so far mild and most likely benign early repolarization pattern (ERP). Early repolarization syndrome is diagnosed in patients resuscitated from sustained polymorphic ventricular tachycardia or ventricular fibrillation without heart disease and an ERP with J-point elevation ≥ 1 mm in adjacent inferior and/or lateral ECG leads [6, 7]. In the majority of cases ERP is a benign finding with a prevalence of up to 10% in the general population but is much more common in idiopathic ventricular fibrillation [8]. It should be noted that the early repolarization pattern is more pronounced over time than in the immediate post-resuscitation situation, although this observation of marked temporal variability was recently confirmed in another study [9]. The recommendation for genetic diagnostics in early repolarization syndrome is rated low by the ESC guideline on sudden cardiac death [6]. Possible mutations associated with early repolarization syndrome are mainly SCN5A [10] or KCND3 mutations [11]. The European Heart Rhythm Association (EHRA)/Heart Rhythm Society (HRS)/Asia Pacific Heart Rhythm Society (APHRS)/Latin American Heart Rhythm Society (LAHRS) Expert Consensus Statement on the state of genetic testing for cardiac diseases [12] does not recommend genetic diagnostics in asymptomatic patients and sees the possibility of diagnostics in patients who have survived sudden cardiac death. As the prognosis of asymptomatic ERP is good, ICD therapy is usually not recommended. In our patient the combination of ERP with intake of a toxic dosage of nicotine most likely caused VF so that we estimated a very low recurrence rate for VF and did not recommend an implantable defibrillator. However, the patient and his wife voted for secondary ICD prevention.

With regard to the management of nicotine intoxication, uptodate.com provides a structured management concept. Apart from the initial patient assessment and decontamination, possibly with activated charcoal, as well as the standardized management of complications, e.g. cholinergic syndromes or arrhythmias according to the respective therapy standards, there is no specific antidote.

## Conclusion

The negative long-term effects of nicotine are well known. Here we present the first case of acute oral intoxication with liquid nicotine that led to ventricular fibrillation and aborted sudden cardiac death in the presence of an electrocardiographic early repolarization pattern.

## Data Availability

No datasets were generated or analysed during the current study.

## Abbreviations

CT: Computertomography
ECG: Electrocardiogram
ERP: Early repolarization pattern
ICD: Implantable cardioverter defibrillator
Kg: Kilogram
Mg: Milligram
MRI: Magnetic resonance imaging
Ms: Milliseconds
SCD: Sudden cardiac death
VF: Ventricular fibrillation
